# Incidence and risk factors of subsyndromal delirium after curative resection of gastric cancer

**DOI:** 10.1186/s12885-018-4681-2

**Published:** 2018-07-27

**Authors:** Heesung Hwang, Kwang-Min Lee, Kyung-Lak Son, Dooyoung Jung, Won-Hyoung Kim, Joo-Young Lee, Seong-Ho Kong, Yun-Suhk Suh, Hyuk-Joon Lee, Han-Kwang Yang, Bong-Jin Hahm

**Affiliations:** 1 Department of Psychiatry, Seoul National University Hospital, Seoul, Korea; 2 Department of Psychiatry and Behavioral Sciences, Seoul National University College of Medicine, 101 Daehak-ro, Jongno-gu, Seoul, 03080 Korea; 3 Public Health and Medical Service, Seoul National University Hospital, Seoul, Korea; 4 Department of Psychiatry, Gyeonggi Provincial Medical Center Uijeongbu Hospital, Uijeongbu, Korea; 5 Department of Human Factors Engineering, Ulsan National Institute of Science and Technology, Ulsan, Korea; 6 Department of Psychiatry, Inha University Hospital, Incheon, Korea; 7 Department of Health Management, Armed Forces Medical Command, Seongnam, Korea; 8 Department of Surgery, Seoul National University Hospital, Seoul, Korea

**Keywords:** Subsyndromal delirium, Incidence, Risk factor, Stomach neoplasm, Gastrectomy

## Abstract

**Background:**

Subsyndromal delirium, a condition in which patients exhibit some, but not all, of the symptoms of delirium, can negatively affect the outcomes of patients with cancer. However, the incidence of subsyndromal delirium in patients with gastric cancer is unknown. Here, we investigated the incidence and risk factors of subsyndromal delirium after curative resection of gastric cancer.

**Methods:**

We recruited consecutive patients with gastric cancer who were scheduled for curative resection at a tertiary hospital. Patients’ subsyndromal delirium symptoms were serially assessed preoperatively and 1, 2, 3, and 7 days postoperatively using the Delirium Rating Scale-Revised-98 (DRS-R-98). A DRS-R-98 score of 8–14 at any postoperative assessment was considered to indicate subsyndromal delirium. Sociodemographic and pre−/intra-operative clinical data were also assessed. Logistic regression analyses were used to determine the associated risk factors.

**Results:**

Data were analysed from 163 out of 217 eligible patients. Postoperative delirium occurred in one patient (0.6%) and subsyndromal delirium occurred in 19 patients (11.7%). Age ≥ 70 years (odds ratio, [OR] 3.85; 95% confidence interval [CI], 1.36–10.92; *p* = 0.011) and education level ≤ 9 years (OR, 3.98; 95% CI, 1.39–11.41; *p* = 0.010) were independent risk factors of subsyndromal delirium after adjusting for preoperative cognitive function. Other pre−/intra-operative variables including anxiety/depression, poor sleep quality, and anaesthesia duration were not associated with subsyndromal delirium.

**Conclusions:**

In contrast to the low incidence of delirium among patients undergoing curative resection of gastric cancer, a substantial proportion of such patients experienced subsyndromal delirium. Considering the prognostic implications, more careful detection and management of subsyndromal delirium may be warranted in patients with gastric cancer.

**Electronic supplementary material:**

The online version of this article (10.1186/s12885-018-4681-2) contains supplementary material, which is available to authorized users.

## Background

Delirium is an acute brain failure syndrome with fluctuating symptoms of inattention, confusion, and disorganized thinking. Delirium commonly occurs postoperatively, with an incidence of up to 73% during the postoperative period and 14–24% during hospital admission [1, 2]. Delirium is also a common complication after cancer resection, occurring in 11–36% of patients with non-gastric cancer [3]. The variability in the incidences of delirium may be related to the characteristics of the patient, disease, and/or treatment [3–6]. Critically, patients who develop delirium have an increased risk of rehospitalisation, have higher mortality and morbidity, and exhibit long-term declines in cognitive function [7–9]. Therefore, diagnosing delirium quickly and accurately is not only important for ensuring patient safety (e.g., preventing falls) [10] and proper treatment [11], but also for anticipating patient prognosis.

Subsyndromal delirium is a partial delirium syndrome or a “pre-delirious” phase and is a milder form of delirium rather than a distinct disease [12]. Indeed, patients with subsyndromal delirium display only a few delirium symptoms (e.g., inattention, thought disturbances, increased vigilance, irritability, anxiety, restlessness, and/or sleep disturbances) without meeting the full criteria of delirium [12, 13]. Subsyndromal delirium has been of clinical interest since the early twenty-first century, and, like delirium, subsyndromal delirium is associated with negative patient outcomes, such as lengthened hospital stays, worse cognitive and functional outcomes, and higher mortality rates [14–18]. Despite its clinical importance, detecting subsyndromal delirium is difficult owing to its fluctuating course and mild symptoms [19]. The incidence of subsyndromal delirium is highly variable, ranging from 0.9 to 36.5% [13]. Moreover, while risk factors for delirium have been identified (including old age, pre-existing cognitive impairment, extensive surgical procedure, longer operation, higher number of comorbidities, blood transfusion, longer management in an intensive care unit, and decreased serum albumin concentration [3, 5, 6, 20–24]), few studies have examined the factors associated with subsyndromal delirium [13, 25], although Cole et al. have suggested that the risk factors of subsyndromal delirium are similar to those associated with delirium [13]. Nevertheless, as subsyndromal delirium may be a marker of underlying medical conditions that are not severe enough to cause full delirium [13], it could be considered that risk factors of subsyndromal delirium could be intrinsic factors in those of delirium.

Compared to studies of patients with other cancers, recent studies of patients with gastric cancer report a lower incidence of delirium in both adults (0.5–6.3%) and the elderly (16.1–31.7%) [26–30]. Although delirium in patients with gastric cancer (or other cancers) is associated with a poor prognosis, few studies have examined the incidence, risk factors, and prognostic effects of delirium in patients with gastric cancer; this may be because of the low incidence of delirium in patients with gastric versus other types of cancer. While it is unclear why gastric cancer is associated with a lower incidence of delirium, the patient- and/or treatment-related characteristics of gastric cancer may be contributing factors. Furthermore, gastric cancer resection may be less deliriogenic compared to the surgical treatments for other cancer types [2, 28].

Although the incidence of delirium in patients with gastric cancer is low, the incidence of subsyndromal delirium in patients with gastric cancer may be even more clinically important, particularly as an indicator of underlying medical conditions, surgical outcomes, or prognosis [15, 31]. Hence, investigating the incidence and risk factors of subsyndromal delirium in patients with gastric cancer is essential.

Here, we prospectively determined the incidence of subsyndromal delirium in patients who were scheduled to undergo curative gastric cancer resection. To investigate the factors associated with an increased risk of developing subsyndromal delirium, we evaluated the patients’ social and medical characteristics, preoperative laboratory data, intraoperative data including surgery- and anaesthesia-related factors, and preoperative psychiatric information.

## Methods

### Patients and procedures

Our target study population included patients admitted to the surgery department who were scheduled to undergo an operation at a tertiary general hospital in Seoul, Republic of Korea, between May 2016 to April 2017. We included patients with gastric cancer who were at least 40 years of age and were scheduled to undergo curative resection and who had adequate Korean literacy to complete study questionnaires. We excluded patients scheduled to undergo surgery for gastrointestinal stromal tumours or for palliative purposes, those with a past history of another cancer, and those experiencing delirium at the time of enrolment. On the day of admission (1–3 days before surgery), the study participants were interviewed and informed about the design and aims of the study. Informed consent was obtained from all of the included participants. This study was approved by the Institutional Review Board of Seoul National University Hospital (IRB No. H-1505-045-671).

### Delirium assessments

A trained research nurse or psychiatrist assessed delirium in patients before surgery and at 1, 2, 3, and 6 to 7 days after surgery. Subsyndromal delirium was assessed using the Delirium Rating Scale-Revised-98 (DRS-R-98). The DRS-R-98 is a clinician-rated scale for assessing delirium that consists of 16 items, and, more specifically, 13 severity items (scores 0–39) and three diagnostic items (scores 0–7) [32]. The severity items, where a higher score indicates worse symptoms, gradually measure the intensity of each delirium symptom, including sleep continuity, orientation and attention, perceptual disturbances, thought disturbances, memory disturbances, and changes in motor activity. The diagnostic items, which were not used in our study, are optional for differentiating delirium from other diagnoses. The DRS-R-98 has been used previously as a screening tool to detect and assess subsyndromal delirium [33–36]. The DRS-R-98 is more sensitive than other tools for detecting the symptoms of subsyndromal delirium, such as mild sleep discontinuity, circumstantial thought processes, and mild distraction [33]. Given that subsyndromal delirium is a less-severe form of delirium rather than a distinct disease, we presumed that a tool with specific severity and cutoff scores would be more appropriate for assessing subsyndromal delirium than other category-based assessments [13, 33]. Consistent with previous studies, we considered DRS-R-98 scores from 8 to 14 at any postoperative assessment to indicate the presence of subsyndromal delirium, while a score of 15 or more indicated full-onset delirium [37, 38].

### Sociodemographic and clinical assessments

The social and medical patient characteristics including age, education, and comorbidities were collected using self-report questionnaires and electronic medical charts. Comorbidities were scored using the International Statistical Classification of Diseases and Related Health Problems, 10th Revision version of the Charlson Comorbidity Index (CCI). Age was excluded from the CCI calculation and analysed independently. The operation and anaesthesia records were used to collect the surgery (surgical method and resection type), anaesthesia (anaesthesia time, main anaesthetic agent, intraoperative analgesic agent), preoperative laboratory, and medication administration data.

In addition, patients’ cognitive function, anxiety and depressive symptoms, and sleep quality were assessed preoperatively. Cognition was assessed using the Korean version of the Mini-Mental State Examination (MMSE) [39]. The MMSE is widely used to screen for neurocognitive disorders, as it measures patients’ orientation, memory registration and recall, attention/calculation, and language abilities [40, 41]. Here, an MMSE score of 23 or below indicated suspicion of neurocognitive dysfunction [42].

Depression and anxiety were self-rated using the Hospital Anxiety and Depression Scale (HADS), a 14-item survey that consists of seven anxiety items and seven depression items [43]. We utilized the classic cutoff score of 8 to indicate anxiety and depression instead of the recently recommended lower cutoffs [44].

Sleep quality was measured using the Pittsburgh Sleep Quality Index (PSQI). Sleep quality is a subjective measurement of the distress that is related to inadequate night-time sleep despite the opportunity to sleep and/or the negative daytime consequences of inadequate night-time sleep [45]. The PSQI assesses various aspects of sleep quality, including the subjective sleep quality, total sleep time, sleep efficiency, sleep disturbances, use of sleep medication, and daytime dysfunction [46]. Considering the characteristics of our study participants, the recommended cutoff threshold of > 8 for patients with cancer was used to indicate poor sleep quality, rather than the cutoff of > 5 that is used for the general population [47].

### Statistical analysis

The pre−/intra-operative and patient−/treatment-related variables are presented as the mean and standard deviation (SD) for continuous variables and as the number of patients and percentage for categorical variables. To compare the patient- or treatment-related characteristics of participants with and without subsyndromal delirium, several statistical analyses were performed. Independent *t*-tests or Wilcoxon-Mann-Whitney U tests were used to compare continuous variables. The Kolmogorov-Smirnov test was applied for normality testing, and either chi-squared tests or Fisher’s exact tests were used to compare categorical variables. We examined the bivariate correlations among the baseline DRS-R-98 score, the highest DRS-R-98 score after surgery, and other continuous variables. For all analyses, *p* < 0.05 indicated a statistically significant difference. Univariate logistic regression analyses were used to examine if each pre−/intra-operative categorical variable was associated with subsyndromal delirium. Variables with a *p*-value of < 0.1 were included in the multivariate logistic regression model. Confounders that were potentially related to the significant variables were included in the model for adjustment. Variables with a *p*-value of < 0.05 from the multivariate logistic regression analyses were considered independent risk factors of subsyndromal delirium. All statistical analyses were conducted using IBM SPSS 23 for windows (IBM Corp., Armonk, NY, USA).

## Results

### Incidence of delirium and subsyndromal delirium

**Fig. 1:**
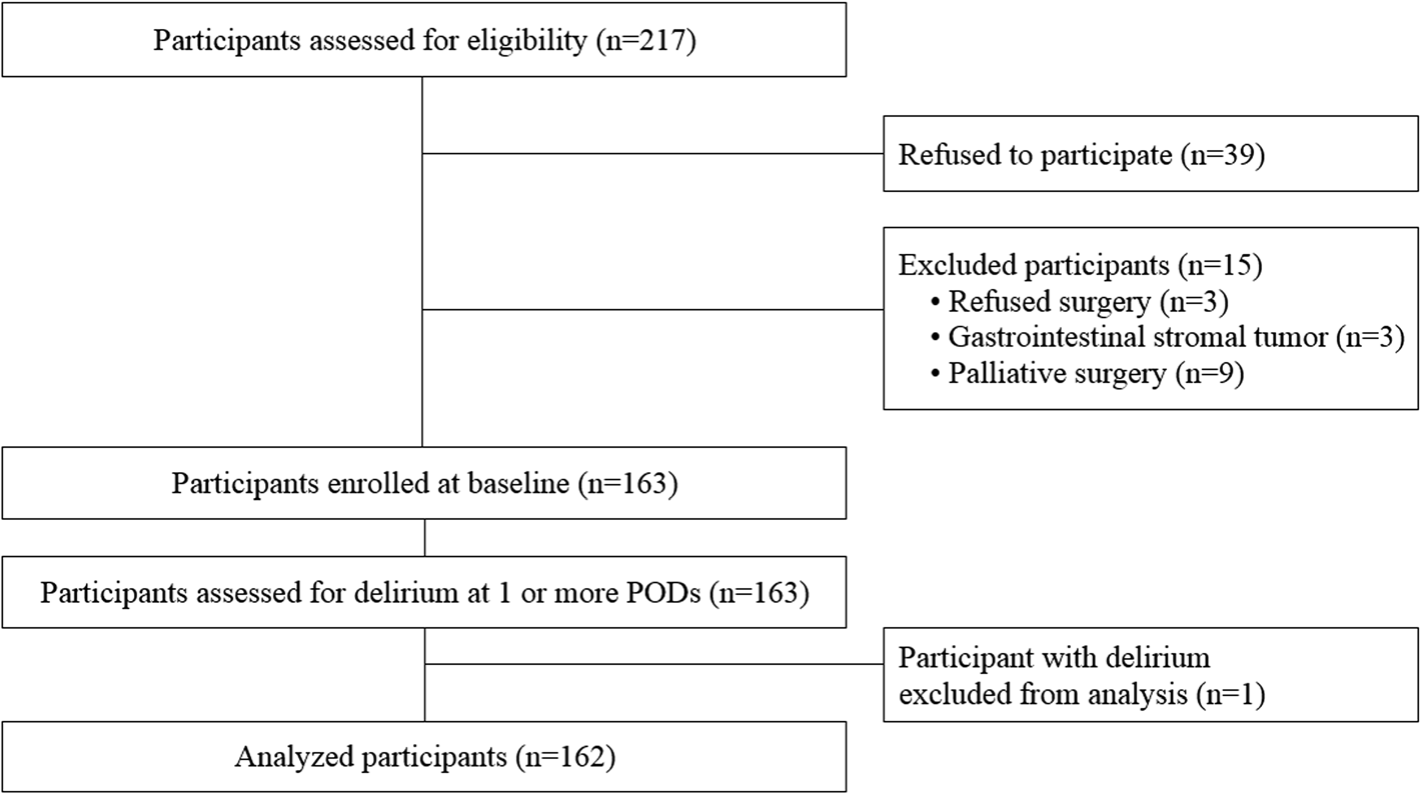
Flow chart summarizing the enrolment process

During the study period, 217 patients were admitted for surgery. After excluding ineligible patients, a total of 163 patients were enrolled in our study. Figure 1 illustrates a flow chart of the enrolment process. Among the participants, one patient had a DRS-R-98 score ≥ 15 after surgery and 21 patients had a score between 8 and 14. Among the patients with postoperative scores of 8–14, two had presented with preoperative DRS-R-98 scores that indicated subsyndromal delirium. The DRS-R-98 scores of these patients did not increase after surgery; thus, they were not considered to have postoperative subsyndromal delirium and were considered to be part of the group with no delirium. Based on the postoperative DRS-R-98 scores, one (0.6%) and 19 (11.7%) patients showed postoperative delirium and subsyndromal delirium, respectively. The patient with full-onset delirium was excluded from subsequent analyses, as we wanted to focus only on patients with subsyndromal delirium symptoms. The DRS-R-98 scores were generally the highest on postoperative day 1 and then gradually decreased (Table 1).

**Table 1:** The DRS-R-98 scores before and after surgery among the patients

	DRS-R-98 (mean ± SD)				
	Preop	POD 1^a^	POD 2^a^	POD 3^a^	POD 7^a^
No delirium (*n* = 143)	1.87 ± 1.67	3.46 ± 1.91	2.74 ± 1.75	2.12 ± 1.92	2.04 ± 1.81
SSD^b^ (*n* = 19)	2.31 ± 1.97	7.74 ± 1.73	6.21 ± 2.12	5.28 ± 3.51	4.00 ± 2.92
Number of patients with newly developed SSD at each POD		14 (73.7%)	2 (10.5%)	2 (10.5%)	1 (5.3%)
Number of patients with a DRS-R-98 score ≥ 8 at each POD	2 (1.2%)^c^	16 (9.9%)	6 (3.8%)	8 (5.1%)	4 (2.7%)

*Abbreviations*: *DRS-R-98* Delirium Rating Scale-Revised-98, *SD* standard deviation, *preop* preoperative, *POD* postoperative day, *SSD* subsyndromal delirium

^a^Missing data were excluded from the analysis (Number of excluded patients: one at POD 1, five at POD 2, six at POD 3, and 16 at POD 7)

^b^Patients with a DRS-R-98 score of < 8 before surgery and a score in the range of 8 to 14 on at least one POD

^c^Two patients with a preoperative DRS-R-98 score of ≥8 were not considered to have SSD

### Sociodemographic and medical characteristics

The sociodemographic and pre−/intra-operative characteristics of patients both with and without subsyndromal delirium are shown in Table 2. Patients with subsyndromal delirium were significantly older than were patients without subsyndromal delirium (70.1 years vs. 61.3 years, *p* = 0.001), and a higher proportion of individuals with subsyndromal delirium than without were aged 70 years or above (57.9% vs. 24.5%, *p* = 0.002). A significantly higher proportion of patients with vs. without subsyndromal delirium had an education level of 9 years or less (63.2% vs. 28.6%, *p* = 0.003). The comorbidity scores and surgical and anaesthetic variables were not significantly different between the two patient groups.

**Table 2:** Demographic and clinical characteristics of patients

Variable	No delirium (*n* = 143)	Subsyndromal delirium (*n* = 19)	
	Mean ± SD or n (%)		*p* value^a^
Age (years)	61.31 ± 10.82	70.11 ± 7.49	0.001^*^
Age ≥ 70	35 (24.5%)	11 (57.9%)	0.002^*^
Male	91 (63.6%)	14 (73.7%)	0.389
Education level, ≤9 years^b^	38 (28.6%)	12 (63.2%)	0.003^*^
Body mass index (cm/kg^2^)	22.55 ± 3.08	23.46 ± 3.12	0.230
> 25	26 (18.2%)	4 (21.1%)	0.756
< 18.5	13 (9.1%)	1 (5.3%)	1.000
CCI (except age)			1.000
2–3	128 (89.5%)	17 (89.5%)	
≥ 4	15 (10.5%)	2 (10.5%)	
Preop midazolam use for anaesthesia-assisted gastroscopy	37 (26.1%)	4 (21.1%)	0.783
Pathological staging			
0^c^	4 (2.8%)	1 (5.3%)	
I	104 (72.7%)	12 (63.2%)	
II	22 (15.4%)	2 (10.5%)	
III	12 (8.4%)	3 (15.8%)	
IV	1 (0.7%)	1 (5.3%)	
Surgical procedure			0.563
Laparoscopic	113 (79.0%)	14 (73.7%)	
Open	30 (21.0%)	5 (26.3%)	
Resection type			
Total gastrectomy	33 (23.1%)	4 (21.1%)	
Distal gastrectomy	77 (53.8%)	13 (68.4%)	
Proximal gastrectomy	4 (2.8%)	0	
Pylorus-preserving gastrectomy	29 (20.3%)	2 (10.5%)	
Anaesthesia time (minutes)	266.47 ± 57.57	287.95 ± 45.03	0.120
Highest quartile	34 (23.8%)	5 (26.3%)	0.780
Main anaesthetic agent			0.767
Sevoflurane or desflurane (inhalants)	112 (78.3%)	16 (84.2%)	
Propofol (TIVA)	31 (21.7%)	3 (15.8%)	
Intraoperative analgesic agent			1.000
Remifentanil	131 (91.6%)	18 (94.7%)	
Fentanyl	12 (8.4%)	1 (5.3%)	

*Abbreviations*: *SD* standard deviation, *CCI* Charlson Comorbidity Index, *preop* preoperative, *TIVA* total intravenous anaesthesia

^a^Continuous variables were analysed with independent *t*-tests, while categorical variables were analysed with chi-squared tests or Fisher’s exact tests

^b^Missing data were excluded from the analysis (10 patients for the education level)

^c^Among the five patients with pathological stage 0, four patients were treated with endoscopic resection before admission for surgery and one patient was treated with neoadjuvant chemotherapy

^*^p < 0.01

### Laboratory and psychiatric variables

We evaluated the patients’ preoperative laboratory data, including the leukocyte count and haemoglobin concentration, as well as the electrolyte, total protein, and albumin levels, and estimated the glomerular filtration rate using the Cockcroft-Gault equation (Additional file 1: Table S1). In previous studies, these data have been associated with delirium [5, 48–50]. Less than 5% of the patients had leukocytosis, serum sodium or potassium abnormalities, or a low albumin level, and none of the patients with subsyndromal delirium had abnormal results for white blood cell, electrolyte, and albumin. The proportions of patients with low haemoglobin levels, low serum protein levels, or an estimated glomerular filtration rate < 60 were 15.4, 8.6, and 42.6%, respectively; no significant differences in the proportions of these measures were observed between the two patient groups.

Although a higher proportion of patients with vs. without subsyndromal delirium had a low MMSE score, the difference between the groups was not significant. The proportions of patients with preoperative psychiatric symptoms, such as anxiety, depression, and sleep quality, did not significantly differ between the groups (Additional file 1: Table S2).

In the bivariate correlation analyses among DRS-R-98 and other continuous variables, the highest score of DRS-R-98 after surgery was significantly correlated with the DRS-R-98 score at baseline, age, education level, anaesthesia time, MMSE at baseline, and PSQI at baseline (Additional file 1: Table S3).

### Risk factors of subsyndromal delirium

Univariate logistic regression analyses revealed that older age (odds ratio [OR], 4.24; 95% confidence interval [CI], 1.58–11.39; *p* = 0.004) and a low level of education (OR, 4.29; 95% CI, 1.57–11.71; *p* = 0.005) were significantly associated with subsyndromal delirium (Table 3). Univariate logistic regression analyses using a continuous covariate showed that these same variables, along with MMSE (OR, 0.89; 95% CI, 0.79–0.99; *p* = 0.037), were significantly associated with subsyndromal delirium (Additional file 1: Table S4). Although preoperative cognitive dysfunction was not significantly associated with subsyndromal delirium, the MMSE score as a baseline state of cognitive function was included in the multivariate model because the MMSE, as a preoperative brain reserve, could have potential interaction with age, education level, and subsyndromal delirium. In the multivariate logistic regression analysis, after adjusting for preoperative cognitive function, both older age (OR, 3.85; 95% CI, 1.36–10.92; *p* = 0.011) and a low education level (OR, 3.98; 95% CI, 1.39–11.41; *p* = 0.010) were identified as statistically significant risk factors of subsyndromal delirium (Table 4). Multivariate logistic regression analyses using a continuous covariate showed that these same variables were statistically significant risk factors (Additional file 1: Table S5).

**Table 3:** Univariate logistic regression analysis to examine the risk factors of subsyndromal delirium

Variable	OR (95% CI)	*p* value
Age, ≥70 years	4.24 (1.58–11.39)	0.004^*^
Male	1.60 (0.55–4.70)	0.392
Education level, ≤9 years	4.29 (1.57–11.71)	0.005^*^
Body mass index		
Body mass index < 18.5	0.58 (0.07–4.80)	0.615
Body mass index ≥18.5, ≤25.0	1.00 (reference)	–
Body mass index > 25.0	1.26 (0.38–4.17)	0.703
CCI (except age), ≥4	1.00 (0.21–4.78)	0.996
Preop midazolam use for anaesthesia-assisted gastroscopy	0.76 (0.24–2.43)	0.639
Pathological stage, ≥II	1.42 (0.50–4.03)	0.505
Surgery procedure type, laparoscopic	0.74 (0.25–2.23)	0.596
Resection type, total	0.89 (0.28–2.86)	0.844
Anaesthesia time, higher quartile	1.15 (0.38–3.41)	0.808
Main anaesthetic agent, propofol (TIVA)	0.68 (0.19–2.48)	0.556
Intraoperative analgesic agent, fentanyl	0.61 (0.07–4.95)	0.640
MMSE, ≤23^a^	2.05 (0.52–8.04)	0.305
HADS anxiety, ≥8^a^	0.77 (0.20–2.88)	0.692
HADS depression, ≥8^a^	0.68 (0.18–2.53)	0.563
PSQI, >8^a^	2.06 (0.50–8.52)	0.317

*Abbreviations*: *OR* odds ratio, *CI* confidence interval, *CCI* Charlson Comorbidity Index, *TIVA* total intravenous anaesthesia, *MMSE* Mini-Mental State Examination, *HADS* Hospital Anxiety and Depression Scale, *PSQI* Pittsburgh Sleep Quality Index

^a^Variables were assessed preoperatively

^*^p < 0.01

**Table 4:** Multivariate logistic regression analysis to determine the independent risk factors of postoperative subsyndromal delirium

Variable	OR (95% CI)	*p* value
Age, ≥70 years	3.85 (1.36–10.92)	0.011^*^
Education level, ≤9 years	3.98 (1.39–11.41)	0.010^**^
MMSE, ≤23	0.95 (0.21–4.36)	0.950

*Abbreviations*: *OR* odds ratio, *CI* confidence interval, *MMSE* Mini-Mental State Examination

^*^p < 0.05; ^**^p < 0.01

## Discussion

Our study is the first prospective observational investigation of the incidence and risk factors of postoperative subsyndromal delirium in patients with gastric cancer. In our study, the incidence of postoperative subsyndromal delirium after curative gastric cancer resection was 11.7%, with older age and low education level being identified as significant risk factors.

Here, the number of patients we identified as having delirium was too small to determine the incidence. Nonetheless, this finding is consistent with the results of previous studies showing an incidence of < 1% for postoperative delirium in patients with gastric cancer [28]. The incidence of postoperative subsyndromal delirium that we observed was also lower than that reported in other studies of patients who underwent cardiac surgery or had head and neck cancer, i.e., 11.7% vs. 32–45% [20, 51, 52]. The lower incidence that we observed may have been because our patients had less comorbidity. We found that 78% of the participants had no preoperative comorbidity outside the primary disease of gastric cancer, as determined using the CCI. Less than 10% of the study participants had comorbidities known to increase the risk of delirium, such as a history of cerebrovascular incidents, poorly controlled diabetes, or marked liver disease [1, 53, 54]. Additionally, the low comorbidity level that we found in our patients has been observed previously in patients with gastric cancer who were scheduled for curative resection [28, 55, 56]. In summary, the relatively low incidence of delirium and subsyndromal delirium after gastric cancer resection could be related to the low comorbidity level, and low comorbidity level may be a characteristic of patients with curative resection of gastric cancer.

In addition to a higher degree of comorbidity, other variables, such as intraoperative factors, laboratory abnormalities, and the use of certain medications, are known to increase the risk of postoperative delirium [3, 5, 6, 20–24]. However, in the present study, we found that many of the risk factors for delirium were not associated with subsyndromal delirium. Our analyses revealed that older age and a low education level were risk factors of postoperative subsyndromal delirium both before and after adjusting cognitive performance. Age is a well-known risk factor for delirium and has been previously identified as a risk factor of subsyndromal delirium [15, 22, 57, 58]. However, it remains unclear whether subsyndromal delirium is affected by old age itself or by the increased comorbidity, lower performance, and lower brain reserves that are associated with aging. Studies have shown that age is a risk factor of delirium, even after adjusting for related comorbidities [59, 60]. Similarly, the results of our study suggest that older age is an independent risk factor for subsyndromal delirium.

In the current study, we also demonstrated that a low level of education was a risk factor of subsyndromal delirium. This finding may be explained by the concept of cognitive reserve, i.e., the ability of the brain to compensate for brain damage, where education is considered one of the proxies [61, 62]. A low education level may imply a lower cognitive reserve; thus, such an individual would be vulnerable to postoperative brain changes and prone to subsyndromal delirium. However, the connection between education level and delirium is unclear. While some studies have reported that education level is correlated with the development of delirium, subsyndromal delirium, preoperative global health status, and postoperative cognitive deficits [23, 58, 63–65], the results of other studies do not indicate that a low level of education is an independent risk factor of delirium [66, 67]. Our results could suggest that old age and a low education level are predisposing factors of postoperative subsyndromal delirium in patients with little comorbidity. Further studies utilizing detailed assessments of subsyndromal delirium and cognitive reserve are needed to determine whether education level is an independent risk factor for subsyndromal delirium in different clinical populations.

The present study has several limitations. First, our sample size was relatively small for identifying the risk factors of subsyndromal delirium, considering the low incidence of subsyndromal delirium in the study population. Some demographic and clinical factors (including BMI, MMSE, anaesthesia time and PSQI) were not statistically significant in logistic regression analysis with categorical variables, which might result from a lack of power in this study. Furthermore, in this study, we did not collect data from an age-matched control group of non-surgical healthy individuals. Therefore, we are unable to compare our data collected from patients with gastric cancer to those from patients who experience subsyndromal delirium but do not have gastric cancer. Second, the symptoms of subsyndromal delirium were not assessed on the day of surgery, possibly resulting in under-diagnosis. However, as information about subsyndromal delirium was collected by asking about symptoms from the most recent 24-h, the likelihood of missing subsyndromal delirium was minimal. Additionally, subsyndromal delirium was assessed based on the highest DRS-R-98 scores obtained during the 7 days following surgery. While DRS-R-98 scores were recorded periodically throughout the first 7 post-operative days, we did not have sufficient data to examine whether any patients showed persistent subsyndromal delirium throughout this period. Third, our study was a single-centre study; therefore, the perioperative risk factors could not be thoroughly investigated. Given that the intraoperative and postoperative management of patients has been associated with the emergence of delirium [21, 53, 68], future studies should investigate different perioperative factors, such as the surgical/anaesthesia protocol, and pain management strategies, as potential risk factors of subsyndromal delirium. Fourth, currently, no established instrument exists for assessing subsyndromal delirium. In line with suggestions from previous studies, this study considered DRS-R-98 scores of 8 to 14 at any postoperative assessment to be indicative of subsyndromal delirium [37, 38]. Histograms showing DRS-R-98 scores at baseline and the highest postoperative DRS-R-98 scores (Additional file 1: Figure S1) may indicate that our data support the use of the recommended DRS-R-98 range for determination of subsyndromal delirium. However, questions remain regarding the reliability of such cut-off scores. Furthermore, in this study, we used logistic regression analysis for categorical variables (including subsyndromal delirium, older age, a low education level, and preoperative cognitive dysfunction) to investigate risk factors of subsyndromal delirium. While the results obtained through examination of these categorical variables may be valuable for understanding clinical implications, they may be biased by the use of cut-off points for continuous variables. In particular, bivariate correlation analyses among continuous variables conducted in this study indicated that anaesthesia time, MMSE, and PSQI, in addition to age and education level, were also significantly correlated with the highest postoperative DRS-R-98 scores. Therefore, future studies are needed to investigate the appropriate range of DRS-R-98 scores for indication of subsyndromal delirium and to examine the association between the symptom domains of DRS-R-98 as continuous variables and other clinical variables. Fifth, selection bias in sample recruitment may have affected the results in this prospective study. The percentage of enrolled patients out of eligible patients was 82.0% (178/217), and the exclusion rate after enrolment was 8.4% (15/178). Finally, our study involved only patients with gastric cancer with little preoperative comorbidity who were scheduled to undergo curative resection. Notably, few comorbidities were identified in our study; this finding may be related to our observation that most of the known risk factors of delirium were not associated with subsyndromal delirium. As such, we caution against generalizing our results to other cancer types or conditions.

Despite these limitations, our study is the first to report the incidence of subsyndromal delirium among patients with gastric cancer. Our findings indicate that although patients who undergo gastric cancer surgery have a relatively low risk of developing full-onset delirium, a significant number of these patients may suffer from subsyndromal delirium. Our data suggest that clinicians should screen for subsyndromal delirium in patients with gastric cancer who are scheduled to undergo curative gastric resection. In particular, patients who are older or who have a low education level may benefit from preoperative preventative and postoperative screening for subsyndromal delirium. Considering the high prevalence of gastric cancer in eastern Asian countries [69, 70], further research is needed on the clinical implications and prognostic significance of subsyndromal delirium in gastric cancer.

## Conclusions

Delirium among patients undergoing curative resection of gastric cancer showed a low incidence of 0.6%, concurrent with previous studies. In contrast, a substantial proportion of such patients experienced subsyndromal delirium with a higher incidence of 11.7%. Older age and low education level were identified as significant risk factors. Considering that subsyndromal delirium has similar prognostic implications to mild delirium, more careful detection and management of subsyndromal delirium may be warranted in patients with gastric cancer.

## Additional file

Additional file 1: **Table S1.** Preoperative laboratory values of participants, **Table S2.** Preoperative psychiatric variables of participants, **Table S3.** Correlations among DRS scores and other continuous variables, **Table S4.** Univariate logistic regression analysis to examine risk factors as continuous variables of subsyndromal delirium, **Table S5.** Multivariate logistic regression analysis to determine the independent risk factors as continuous variables of postoperative subsyndromal delirium, **Figure S1.** Histogram of pre-op DRS and post-op DRS. (DOCX 44 kb)

## Abbreviations

CCI: Charlson Comorbidity Index
CI: Confidence interval
DRS-R-98: Delirium Rating Scale-Revised 98
HADS: Hospital anxiety and depression scale
MMSE: Mini-mental status exam
OR: Odds ratio
PSQI: Pittsburgh Sleep Quality Index
