# Supplementary material for: Incidence and risk factors of subsyndromal delirium after curative resection of gastric cancer
Source: BMC Cancer. 2018 Jul 27;18:765. doi: 10.1186/s12885-018-4681-2 (PMC6062877; doi:10.1186/s12885-018-4681-2)
Supplement: Supplementary file 1 — Table S1. Preoperative laboratory values of participants, Table S2. Preoperative psychiatric variables of participants, Table S3. Correlations among DRS scores and other continuous variables, Table S4. Univariate logistic regression analysis to examine risk factors as continuous variables of subsyndromal delirium, Table S5. Multivariate logistic regression analysis to determine the independent risk factors as continuous variables of postoperative subsyndromal delirium, Figure S1. Histogram of pre-op DRS and post-op DRS. (DOCX 44 kb) [file 12885_2018_4681_MOESM1_ESM.docx]

**Table S1.** Preoperative laboratory values of participants

| Variables | No delirium  (n=143) | Subsyndromal delirium  (n=19) | *p*-value^†^ |
| --- | --- | --- | --- |
| Leukocyte count (10 ^9^/L) | 6.48±2.29 | 6.45±1.67 | 0.951 |
| Leukocytosis (>10.0) | 7 (4.9%) | 0 | 1.000 |
| Hb concentration (g/dL) | 13.60±1.84 | 13.75±1.91 | 0.750 |
| Low Hb concentration  (male <13.5, female <12.0) | 22 (15.4%) | 3 (15.8%) | 1.000 |
| Serum sodium (mmol/L) | 141.23±2.37 | 141.84±1.38 | 0.112 |
| High serum sodium (>145.0) | 6 (4.2%) | 0 | 1.000 |
| Low serum sodium (<135.0) | 1 (0.7%) | 0 | 1.000 |
| Serum potassium (mmol/L) | 4.26±0.36 | 4.19±0.24 | 0.434 |
| High serum potassium (>5.5) | 1 (0.7%) | 0 | 1.000 |
| Low serum potassium (<3.5) | 1 (0.7%) | 0 | 1.000 |
| Total protein (g/dL) | 7.22±0.65 | 7.09±0.48 | 0.389 |
| Low total protein (<6.0) | 12 (8.4%) | 2 (10.5%) | 0.670 |
| Albumin (g/dL) | 4.18±0.40 | 4.16±0.34 | 0.879 |
| Low albumin (<3.3) | 5 (3.5%) | 0 | 1.000 |
| Estimated GFR (mL/min) | 65.48±17.36 | 60.53±13.32 | 0.233 |
| Low eGFR (<60) | 60 (42.0%) | 9 (47.4%) | 0.654 |

^Abbreviations: SD, standard deviation; Hb, haemoglobin; GFR, glomerular filtration rate^

^† continuous variables were analysed with independent T-test, categorical variables were analysed with chi-square test or Fisher's exact test^

**Table S2.** Preoperative psychiatric variables of participants

| Variables | Mean±SD or N (%) | | *p*-value^†^ |
| --- | --- | --- | --- |
| MMSE | 27.42±2.92 | 25.63±5.11 | 0.151 |
| MMSE ≤23 | 12 (8.4%) | 3 (15.8%) | 0.389 |
| HADS anxiety^‡^ | 5.36±3.89 | 5.40±3.42 | 0.968 |
| HADS anxiety ≥8^‡^ | 33 (24.6%) | 3 (20.0%) | 1.000 |
| HADS depression^‡^ | 5.14±3.41 | 4.44±3.18 | 0.433 |
| HADS depression ≥8^‡^ | 34 (25.4%) | 3 (18.8%) | 0.762 |
| PSQI^‡^ | 5.68±3.10 | 7.00±4.17 | 0.194 |
| PSQI >8^‡^ | 18 (15.4%) | 3 (27.3%) | 0.387 |

^Abbreviations: SD, standard deviation; MMSE, Mini-Mental State Exam, HADS, Hospital Anxiety and Depression Scale, PSQI, Pittsburgh Sleep Quality Index^

^† continuous variables were analysed with independent T-test, categorical variables were analysed with chi-square test or Fisher's exact test^

^‡ missing data was excluded from analysis (14 HADS, 34 PSQI)^

| **Table S3.** Correlations among DRS scores and other continuous variables | | | | | | | | | | | |
| --- | --- | --- | --- | --- | --- | --- | --- | --- | --- | --- | --- |
| Variables | Mean±SD | 1 | 2 | 3 | 4 | 5 | 6 | 7 | 8 | 9 | 10 |
| 1. Post-op DRS^†^ | 4.60±2.34 | 1 |  |  |  |  |  |  |  |  |  |
| 2. Pre-op DRS | 1.92±1.70 | 0.33^**^ | 1 |  |  |  |  |  |  |  |  |
| 3. Age | 62.34±10.85 | 0.29^**^ | 0.20^*^ | 1 |  |  |  |  |  |  |  |
| 4. Education level (yr) | 4.34±1.85 | -0.20^*^ | -0.21^*^ | -0.33^**^ | 1 |  |  |  |  |  |  |
| 5. BMI | 22.66±3.09 | 0.15 | 0.01 | 0.02 | <0.01 | 1 |  |  |  |  |  |
| 6. Anaesthesia time | 268.99±56.55 | 0.21^**^ | 0.01 | 0.13 | -0.06 | 0.12 | 1 |  |  |  |  |
| 7. MMSE | 27.22±3.29 | -0.31^**^ | -0.50^**^ | -0.38^**^ | 0.46^**^ | -0.04 | -0.11 | 1 |  |  |  |
| 8. HADS anxiety^‡^ | 5.36±3.83 | 0.10 | 0.23^**^ | -0.08 | 0.13 | 0.02 | 0.07 | 0.10 | 1 |  |  |
| 9. HADS depression^‡^ | 5.07±3.39 | 0.07 | 0.23^**^ | -0.01 | 0.06 | -0.08 | -0.01 | 0.10 | 0.59^**^ | 1 |  |
| 10. PSQI^‡^ | 5.80±3.20 | 0.21^*^ | 0.21^*^ | -0.11 | -0.07 | 0.05 | 0.21^*^ | -0.06 | 0.38^**^ | 0.32^**^ | 1 |
| Abbreviations: SD, standard deviation; DRS, Delirium Rating Scale; MMSE, Mini-Mental State Exam, HADS, Hospital Anxiety and Depression Scale, PSQI, Pittsburgh Sleep Quality Index The table presents the Spearman's rho coefficients for regression variables. ^†^ highest DRS score after surgery ^‡^ missing data was excluded from analysis (14 HADS, 34 PSQI)  ^*^p < 0.05 and ^**^p < 0.01 | | | | | | | | | | | |

**Table S4.** Univariate logistic regression analysis to examine risk factors as continuous variables of subsyndromal delirium

| Variable | OR (95% CI) | *p* value |
| --- | --- | --- |
| Age | 1.09 (1.03–1.15) | 0.002^**^ |
| Education level (year) | 0.60 (0.44–0.81) | 0.001^**^ |
| Body mass index | 1.10 (0.94­­–1.29) | 0.229 |
| Anaesthesia time, higher quartile | 1.01 (1.00–1.01) | 0.124 |
| MMSE^a^ | 0.89 (0.79–0.99) | 0.037^*^ |
| HADS anxiety^a^ | 1.00 (0.87–1.15) | 0.968 |
| HADS depression^a^ | 0.94 (0.80–1.10) | 0.432 |
| PSQI^a^ | 1.12 (0.94–1.34) | 0.196 |

Abbreviations: OR, odds ratio; CI, confidence interval; MMSE, Mini-Mental State Examination; HADS, Hospital Anxiety and Depression Scale; PSQI, Pittsburgh Sleep Quality Index.

^a^Variables were assessed preoperatively.

**p* < 0.05; ***p* < 0.01.

**Table S5.** Multivariate logistic regression analysis to determine the independent risk factors as continuous variables of postoperative subsyndromal delirium

| Variable | OR (95% CI) | *p* value |
| --- | --- | --- |
| Age | 1.07 (1.01–1.14) | 0.023^*^ |
| Education level (year) | 0.65 (0.47–0.90) | 0.009^**^ |
| MMSE | 1.00 (0.87–1.15) | 0.993 |

Abbreviations: OR, odds ratio; CI, confidence interval; MMSE, Mini-Mental State Examination.

**p* < 0.05; ***p* < 0.01.

A. B.


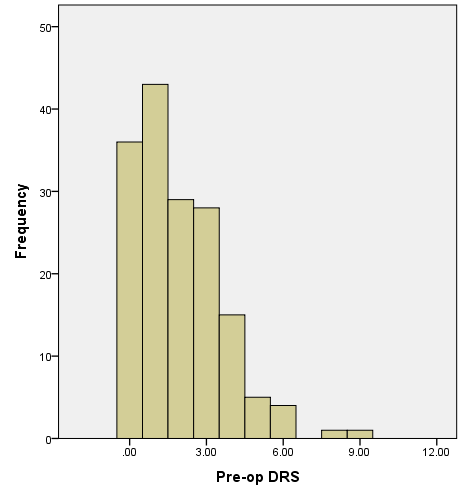

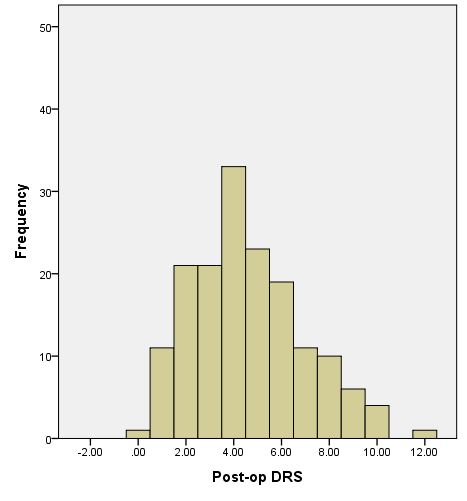


**Figure S1.** Histogram of pre-op DRS and post-op DRS. Abbreviation: DRS, delirium rating scale. Post-op DRS indicates highest DRS score after surgery.
